# Pharmacoepidemiology: An Overview

**DOI:** 10.3390/jcm12227033

**Published:** 2023-11-10

**Authors:** Mònica Sabaté, Eva Montané

**Affiliations:** 1 Department of Clinical Pharmacology, Hospital Universitari Vall d’Hebron, Clinical Pharmacology Research Group, Vall d’Hebron Research Institute, 08035 Barcelona, Spain; monica.sabate@vallhebron.cat; 2 Department of Pharmacology, Therapeutics and Toxicology, Universitat Autònoma de Barcelona, 08193 Bellaterra, Spain; 3 Department of Clinical Pharmacology, Hospital Universitari Germans Trias i Pujol, 08916 Badalona, Spain

**Keywords:** pharmacoepidemiology, effectiveness, pharmacovigilance, regulatory decision, adverse drug reaction, drug safety, patient safety

## Abstract

The aims of this review are to provide a comprehensive overview of the definition and scope of pharmacoepidemiology, to summarize the study designs and methodologies used in the field, to discuss the future trends in the field and new methodologies to address bias and confounding, and finally to give some recommendations to clinicians interested in pharmacoepidemiologic research. Because drug efficacy and safety from randomized clinical trials do not reflect the real-world situation, pharmacoepidemiological studies on drug safety monitoring and drug effectiveness in large numbers of people are needed by healthcare professionals and regulatory institutions. We aim to highlight the importance of pharmacoepidemiologic research in informing evidence-based medicine and public health policy. The development of new designs and methodologies for the generation of valid evidence, as well as new initiatives to provide guidance and recommendations on how to incorporate real-world evidence into the drug development process, are reported on. In addition, we have touched on the implication of artificial intelligence in the management of real-world data. This overview aims to summarize all important aspects to consider when conducting or interpreting a pharmacoepidemiologic study.

## 1. Introduction

Pharmacoepidemiology was born at the end of the last century with the need to assess drug effects due to their massive use in clinical practice. Its purpose is to analyze drug effectiveness and adverse drug reactions (ADRs), to assess patterns of drug use, and to compare the data obtained from clinical trials with the use of drugs in clinical practice [1,2].

The benefits or the efficacy of marketed drugs have been demonstrated through randomized clinical trials (RCT), which are widely accepted to be the gold standard in demonstrating efficacy as the use of randomization, and blinding allows us to avoid biases [3]. However, RCT are conducted in an experimental setting with controlled conditions and selected patients, which do not reflect real-world situations [4,5,6]. Consequently, the efficacy of a drug in clinical trials is often higher than its effectiveness in real clinical practice [7]. The main reasons why those differences appear are related to the low external validity of clinical trials. First, the number of patients who have received the drug is low and selection has strict inclusion criteria in terms of age, gender, co-morbidities, and concomitant medicines. Second, drug safety is usually a secondary endpoint of clinical trials [7]. Third, when the outcome or the exposure is rare or appears after a long period of time, the risk estimation can be underpowered [4,5]. All these reasons highlight the need for post-marketing follow-up of drug and pharmacoepidemiologic studies [2,8]. In the field of pharmacovigilance, where the approach is to collect and analyze safety information related to drugs, the major contribution of pharmacoepidemiology is the use of studies for drug safety monitoring and the identification of unexpected or rare ADRs [9,10]. The safety profile of a drug when it comes to the market is not always well known since clinical trials often include small sample sizes [9] and are evaluated in a short period of time compared to the massive consumption of drugs by the population and the long duration of treatments in real life [2]. Severe safety issues detected in real clinical practice are the most frequent cause of withdrawal of marketed drugs by regulatory authorities [11].

This paper provides a comprehensive overview of the definition and scope of pharmacoepidemiology, as well as a summary of several study designs and methodologies employed in the field. In addition, future trends in the field and the available new methodologies are briefly addressed. We aim to highlight the importance of pharmacoepidemiologic research in informing evidence-based medicine and public health policy. Considering that the JCM is a journal written for clinicians who may not be as familiar with pharmacoepidemiology, this article is intended to be a summarized and understandable review for reading and application by these physicians and other healthcare professionals.

## 2. Pharmacoepidemiology Definition and Objectives

Pharmacoepidemiology is a multifaceted discipline that combines the principles of both pharmacology and epidemiology for the study of the use and effects of drugs in extensive populations. It applies epidemiological methods to the area of clinical pharmacology.

The main objective of pharmacoepidemiology is to enhance the understanding of the benefits and risks associated with drug use, ultimately informing clinical decision making and public health policy [2]. Pharmacoepidemiology has primarily been concerned with postmarketing drug surveillance; however, in recent times, the scope of interest for pharmacoepidemiologists has significantly expanded. Another goal of pharmacoepidemiology is to assess the economic impact and health advantages arising from unintended drug effects [12].

**Figure 1:**
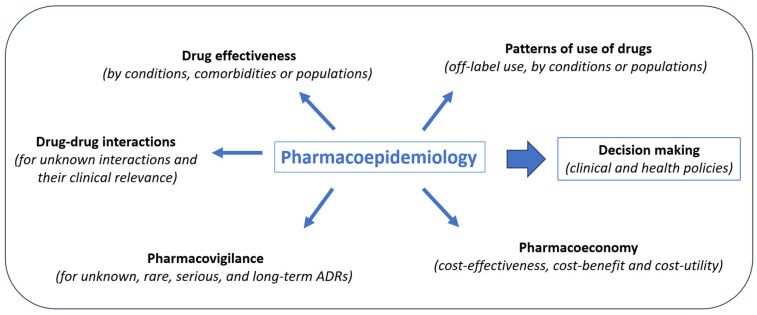
Main goals of pharmacoepidemiology. ADRs: adverse drug reactions.

Indeed, pharmacoepidemiology can offer insights that cannot be obtained through premarketing trials. Pharmacoepidemiologic analysis allows us to compare drug effects in populations of patients not previously assessed such as pediatrics, elderly or pregnant women; to identify patterns of use of drugs in specific diseases or populations; to examine how the effects of drugs are altered by the presence of other drugs (drug–drug interactions) or diseases (co-morbidities); to quantify serious ADRs, to discover rare and unknown ADRs, the effects of a drug overdose, adverse teratogenic effects, or delayed side effects; as well as to compare the costs and outcomes of drugs used for the same illness [12,13]. Some of the differences in effectiveness or safety problems may be explained by pharmacokinetic, pharmacodynamic, or pharmacogenomic characteristics. All of this real-world evidence (RWE) is invaluable in informing healthcare policymakers when deciding on the regulatory aspects of medicines (Figure 1).

## 3. Studies in Pharmacoepidemiology

As mentioned above, pharmacoepidemiologic studies can help to identify the risks and benefits of drugs and inform decisions related to drug use. The selection of a study design depends on the specific research question and available data, mitigating the risk of bias. Generally, large, or extremely large numbers of individuals are included in such studies helped considerably by having access to databases [8].

The field of pharmacoepidemiology includes various types of observational studies, each with its own unique strengths and limitations. Observational studies can be categorized into two main types: descriptive and analytical. Descriptive studies do not have control groups. They just describe one or more variables in a specific population. Analytical studies are studies with control groups, allowing us to make comparisons between them. Descriptive studies include cross-sectional studies, drug utilization studies, and ecological studies. Analytic studies include cohort studies, case–control studies, and other variations that originate from these designs [14]. Some of the main designs are described below and summarized in Table 1.

Cohort studies: Prospective and retrospective cohort studies follow a defined population over time who is exposed to a drug and compare their outcomes (efficacy or safety) with a group of individuals who are not exposed. This study design is useful for studying long-term drug effects and can provide information on the incidence of drug-related outcomes and to explore multiple outcomes associated with a specific exposure. The disadvantages are the need for a large sample size and an extended study period to assess infrequent outcomes, although electronic healthcare databases allow us to analyze large cohorts retrospectively [15,16].

Case-control studies: Case-control studies compare individuals who have a specific outcome (or ADRs) to individuals who do not have that outcome (matched controls), enabling the assessment of the relationship between drug exposure and the specific outcome under investigation. The study then looks at whether the individuals with the outcome were exposed to a drug. This design is useful for studying rare outcomes or diseases and their association with multiple exposures or risk factors. However, one of the primary challenges in case–control studies is the accurate selection of control subjects who are not influenced by exposure or other pertinent risk factors. Additionally, it can be challenging to investigate uncommon exposures, as it would require a substantial number of cases and controls [15,16,17].

Nested case–control studies: Nested case–control studies are a type of study in which cases (all individuals with a specific outcome or ADR) are identified within a larger cohort, and controls (individuals without the outcome) are randomly selected from within the cohort. This design is useful for studying rare outcomes or diseases [16,17].

Case-only design: Case-only designs involve only the cases as subjects. These designs minimize confounding factors by utilizing the exposure and outcome history of each case as a control, thereby removing confounding factors that remain constant over time. They are particularly well-suited for investigating brief exposures in relation to immediate outcomes. Some examples include the case-crossover designs, the self-controlled case series, and the self-controlled risk interval [16,18].

Cross-sectional studies: Cross-sectional studies measure drug use and health outcomes at a single point in time. The data collected at a specific time point may encompass both information on exposure and outcomes. This study can provide a snapshot of drug use and its effects on a population [17].

Drug utilization studies: These studies describe the patterns of utilization of specific groups of drugs according to geographic regions and time. This type of study is valuable for estimating the number of patients who have been exposed to specific drugs within a defined time frame. Incidences and prevalences can be calculated for specific diseases. Additionally, they can provide estimates regarding the extent of rational drug utilization (over- or underutilization) and compliance with established guidelines [14].

Ecological studies: They are also called the analysis of secular trends. In ecological studies, the unit of analysis is typically the entire population, rather than individual subjects, and the frequency of drug use or disease across different geographic or temporal areas is compared. This study design is useful for identifying the patterns of drug use and disease occurrence [19].

Case-population studies: Case-population studies represent a type of ecological study where cases are compared with an aggregated group derived from population data. This study design is commonly employed in pharmacovigilance for the purpose of identifying potential signals and monitoring drug safety [16,20].

Meta-analysis of observational studies (alone or in combination with RCTs): This kind of evidence is desirable and is increasingly being published [21]. The meta-analysis of observational studies is useful in studying rare ADRs, determining the effectiveness of new indications of existing therapies or among subgroups of patients, as well as shortening the time of implementing a new therapy [14].

Pharmacoeconomic studies: Cost–benefit, cost-effectiveness, and cost–utility analysis are used in pharmacoeconomic research. Cost–benefit analysis compares the cost of a drug to its benefits, with all measures in monetary terms, whereas cost-effectiveness analysis compares the costs in monetary terms to the effectiveness measured in clinical terms (such as complications prevented, diseases cured, or the number of lives saved). In cost–utility analysis, the cost of an intervention is linked to the quantity of quality-adjusted life years (QALYs) that can be obtained via the implementation of the said intervention [14].

Target trial emulation: This new study design has recently emerged using observational data to emulate a target trial, aiming to estimate the causal effect of an intervention and minimize biases common to observational studies [16]. The initial step in this approach involves designing a hypothetical and ideal randomized trial, referred to as the “target trial,” which would provide answers to the research question. This target trial is precisely outlined, encompassing all the design aspects, including eligibility criteria, treatment strategies, allocation procedures, follow-up, outcome measurement, causal comparisons, and the plan for analysis. In the second step, the researcher determines the most effective way to reproduce the design components of the target trial using the existing observational data, taking into account various analytical methods and the inherent limitations of observational settings [17,22,23].

Adherence studies: The aim of adherence studies is to use different methodologies to study the process by which patients take their medications as prescribed. Adherence comprises three elements: initiation of therapy, implementation of the dosing regimen, and persistence with treatment. Many systems are available to measure adherence but the big electronic databases again offer us an opportunity to measure a large number of individuals, especially those that have information on drug prescription and dispensation [24]. Several indicators are available to measure adherence such as the Proportion of Days covered (PDC) calculated as the total number of days covered by the dispensation (numerator) divided by a fixed time interval usually defined as 365 days following the treatment initiation [25]. Other indicators can be used, such as the discontinuation rate, cessation rate, and medication refill adherence (MRA) [26]. In addition, more sophisticated methodologies, such as group-based trajectories models (GBTM), allow us to study what the behavior is, regarding adherence of the patients considering their clinical characteristics (sex, age, comorbidities, polypharmacy, etc.) [27]. Group-based trajectory models represent an additional method for summarizing long-term medication adherence that considers the dynamic maturation of adherence over time, identifying patients with similar adherence patterns [28].

The research on the use of drugs that involves the study of doses is not yet optimal since most of these databases do not contain information on the regimen and we can only make an approximation using the calculation of Defined Daily Dose (DDD).

## 4. Sources of Data Collection

To conduct observational studies and obtain data, either primary data collection or secondary data sources are needed. Automated databases containing healthcare data from routine clinical practice are the paradigm of the sources of data collection.

We would like to highlight that data sources should be based on International Health Terminology Standards, that is, codes elaborated by international organizations (such as the WHO) to ensure full interoperability of the data. Consequently, data will be comparable to other studies and by different countries. For example, drugs should be coded with the Anatomical Therapeutic Chemical (ATC) classification System [29], diseases with the International Classification of Diseases and Related Health Problems (ICD) [30], and adverse effects with the MEdDRA dictionary [31].

Considering the possibilities that the big clinical databases offer us in order to research into pharmacoepidemiology, it is important to be sure of the quality and validity of the data. Accuracy and validity, dependability, completeness, legibility, timeliness, and accessibility are some of the elements that make up good data. In pharmacoepidemiologic studies, the value of a database must be evaluated by checking the proportion of individuals correctly classified as exposed, the completeness of individual registrations, the comprehensiveness of information registered, the size of the data source (population coverage), the registration period, accessibility, availability, and cost, the data format (e.g., available age categories), and record linkage (the process of linking records). Furthermore, having information on the data-generating process helps with data interpretation [32].

We describe the main sources of secondary data defined as data routinely gathered for a different purpose such as research. The authors propose herein the use of certain sources of data collection depending on the type of study (Table 1).

1. Electronic health records (EHRs): This refers to electronic versions of a patient’s medical history and health-related information They can provide a wealth of information on medication prescribed, diagnoses, allergies, lab results, radiology images, and health outcomes [6].
2. Prescription databases: These databases typically contain detailed information on medications prescribed to patients such as the drug name, dosage, duration of the prescription, and the prescribing healthcare professional, and can be used to identify patterns of medication use and potential drug interactions [33]. However, in patients with poor adherence to treatment, such information would not be equivalent to the medications taken by the patient. Therapeutic regimens and diary doses usually taken cannot be extracted, limiting the analysis of these data.
3. Pharmacy dispensing databases: These provide confirmation that the patient has acquired the medication and give information about medication adherence, without guaranteeing that the medications have been taken. Therefore, it is a vague indicator of an ambulatory patient’s exposure to a drug [32].
4. Disease registries: These databases collect information on patients with specific medical conditions and can be used to study the effects of drugs on these populations [34].
5. Pharmacovigilance databases: These contain information on suspected ADRs, suspected drugs, and patient outcomes, which are collected from a variety of sources, including healthcare providers, national authorities, pharmaceutical companies, medical literature, and directly from patients [35].
6. Insurance claims databases: These contain information on medical claims and can be used to identify patterns of medication use and adverse events [36].
7. National health surveys: These are large-scale surveys that collect information on the health status, healthcare utilization, and medication use of individuals in a population [32,37].
8. Economic assessment: This calculates the costs of medical care, which includes costs of preparation, administration, monitoring drugs and treating ADRs (including length of stay and monitoring tests performed), and the economic consequences of the benefits of a drug [14].
9. Patient-generated health data: This source has emerged in recent years and is becoming more common due to the digitization of the population with wearable devices and mobile apps. Data can be obtained at short intervals or continuously and can be transmitted to clinicians and researchers. Some examples of these data are glucose blood levels, heart rate, stress level, time and type of physical activity, and hours and quality of sleep per day [38].
10. Social media: Recent evidence has shown that data from social networks such as Facebook or Twitter provide useful information for drug safety analysis [39].
11. There are other specific registries, such as death certificates from national registry databases [40].

## 5. Measures to Avoid Biases and Confounding Factors

The increasing use of secondary data sources has led to the need to use different tools to deal with possible biases. In the following paragraph, several methods to avoid bias are described. In parallel, it is paramount to develop and improve methodologies and statistical methods to address bias and confounding, which are one of the main issues in observational studies. Bias is defined as the result of a systematic error during the design or execution of a study that tends to make the study results different from the true results. This affects the study's validity because the biased results are not valid and produce an erroneous result on average [41].

It is important for pharmacoepidemiology research, when planning, conducting, or evaluating studies to consider all the possible biases. These include selection bias such as prevalent user bias, confounding, and misclassification. It is also important to remember competitive outcomes. In addition, with the use of secondary databases, other biases have to be taken into account, apart from the biases mentioned above, such as the immortal-time bias and the immeasurable time bias, which can be dealt with either in the design or analysis step [42]. The immortal time bias occurs when using electronic databases, and the exposure occurs at a different time than the inclusion of the individual in the cohort, so there is a period in which the individual cannot present the outcome by design. The immeasurable time bias refers to the period under study, during which a subject cannot be recognized as being exposed because of hospitalization or other issues [42]. These two biases are the most common, mainly when using electronic databases, although other types of bias may exist.

Classical restriction, stratification, and multivariable regression to deal with selection bias and confounding have been used, but other methods have been developed for mitigating the biases associated with the study population.

The propensity score (PS) that is used to control the confounding allows us to classify the subjects, taking into account the covariates of interest, into the probability of being treated or not. This allows us to use the four major methods for using PS: matching, stratification, covariate adjustment, and inverse probability of treatment weighting [43]. An extension of the propensity score is the high-dimensional propensity score (HDPS), which allows us to use hundreds of covariates to adjust for confounding, measured before the treatment initiation providing more precise information on the degree of severity of co-morbidities [44]. As for the calculation of the PS, we assume that the covariates do not vary over time. Other methodologies allow us to consider variation over time, whereas other methods permit us to control time-varying confounders in nondynamic treatments (marginal structural models) or time-varying treatments (G-estimation) [17]. The disease risk scores also allow us to classify the subjects using the probability of a specific disease score considering the covariate of interest. The use of observational studies to estimate effectiveness has also led to the development of other methodologies. The instrumental variables method is a useful approach to address uncontrolled confounding in comparative confounding [45]. An instrumental variable is a factor that is variable (the instrument) that is related to treatment but is not related to the study outcome. It involves the following assumptions: (1) it should have an impact on treatment or be associated with treatment due to a shared cause; (2) it should be a factor that is as good as randomly assigned as possible, meaning that it is unrelated to the patient characteristics; and (3) it should be connected to the outcome only through its association with treatment [45].

Other methodologies are useful in dealing with the protopathic bias (when believing a factor to be a consequence of an exposure when, in reality, it is a factor that determines the exposure) such as the lag-time approach consisting of excluding the period previous to the outcome as exposure [46]. To deal with unmeasured confounders, the rule-out approach allows us to estimate the magnitude of the measured confounder to find no association and ‘rules out’ the association with an unmeasured confounder [47].

Borrowing methodologies from biological laboratory experiments, we can use ‘negative control’, studies designed to detect both suspected and unsuspected sources of spurious causal inference. Applied to pharmacoepidemiology, negative controls help to identify and resolve confounding as well as recall bias and other sources of error. We distinguish two types of negative controls: exposure controls and outcome controls. When using this methodology, the researcher has to have expertise in the subject for the choice of negative controls [48].

All of these measures are summarized in Table 2.

## 6. Using Observational Data for Regulatory Decisions

Regulatory agencies need data and studies to help with their decisions. For that reason, some initiatives coming from the European Medicines Agency (EMA) have been set up such as Heads of Medicines Agencies-European Medicines Agency (HMA/EMA) Joint Big Data Taskforce and the Initiative for patients’ registries. The purpose of the HMA/EMA Joint Big Data taskforce was to provide a regulatory overview of the big data landscape so that the EU regulatory framework could be prepared to handle, evaluate, and comprehend this data [49].

However, patient registries are an important source of information for supporting regulatory decision making regarding medicinal products, but not all of them routinely and systematically collect information on adverse events and ADRs [50].

Even though the origin of medicine safety monitoring can be traced back to the creation of systems for patients and healthcare providers to spontaneously report suspected ADRs, it has long been recognized that it is crucial to use all available data, including observational studies. In this regard, the EMA initiated contacts through the International Society for Pharmaceutical Engineering (ISPE) with different centers of pharmacoepidemiology and pharmacovigilance to provide expertise in this field. This resulted in the creation of the European Network of Centers for Pharmacoepidemiology and Pharmacovigilance (ENCePP), which was established in 2007 with the inclusion of over seventy participants. The ENCEPP’s founding principles include transparency, scientific independence, and adherence to common quality standards [51].

After the creation of the ENCePP, in 2021, a larger-scale project known as DARWIN (Data Analysis and Real-World Interrogation Network) was started by the EMA. The goal is to bring together data providers, pharmacoepidemiologists, statisticians, and medical professionals so that EMA and national agencies can use these data whenever needed over the course of medicine’s lifecycle. The DARWIN EU Project provides sources of high-quality, validated real-world data (RWD) on the uses, safety, and efficacy of medicines. It expands and establishes a catalogue of observational data sources for use in medicine regulation. It also addresses specific questions by conducting high-quality, non-interventional studies, which include creating scientific protocols, examining pertinent data sources, and interpreting and reporting study results [52].

Data from networks of databases, frequently spanning multiple countries, are used in an increasing number of pharmacoepidemiologic studies. Combining data from several databases allows for a better understanding of how broadly applicable the findings are. The use of a high number of patients helps to increase accuracy. The possibility of making cross-national comparisons (across countries or geographical regions) allows us to find differences in outcome rates. An additional advantage is that it is quicker to obtain data from a high sample size [17].

Postmarketing studies carried out in population-based databases frequently include data on millions of patients, but they may still be underpowered if the subgroup’s effects are of interest or if the outcomes or exposure of interest are uncommon. Several databases combined could offer the necessary statistical power. A multi-database study (MDS) employs two or more healthcare databases that are not individually linked to one another. Analyses are conducted concurrently on each database using a shared study protocol. Despite the fact that there have been a lot of MDSs performed in Europe over the last ten years, little is known about the specifics and consequences of the current methods for performing them [53]. Five different models are available to conduct multiple data source studies, depending on the application of a standard protocol, the use of unique or shared programs for data extraction, and the application of a standard data model for analysis [53].

Artificial intelligence (AI) has a useful role to play in helping pharmacovigilance. AI, such as ChatGPT or GPT4, can identify adverse effects based on real data from social networks [54,55]. Many initiatives have been taken to try to study the use of IT tools for natural language processing to identify adverse events AI-based medical record review opens up a multitude of possibilities to detect and monitor adverse effects [56,57,58].

Furthermore, the use of observational data for safety regulatory decisions is useful along with observational studies that can be an alternative when evaluating medicines’ effectiveness. The use of RCT often has limitations, such as ethical issues, data generalizability, and difficulty in recruiting patients mainly of rare diseases. The usefulness of real-world evidence for regulatory decision making is increasing, and regulatory agencies such as the Food and Drug Administration (FDA) and EMA have taken the initiative to produce guidance and recommendations on how to incorporate real-world evidence (RWE) into the drug development process [59,60,61].

Additionally, the FDA has a long history of monitoring and assessing the postmarket safety of approved medications using RWD and RWE. RWE has historically been used, albeit less frequently, to support effectiveness. Improvements in RWD accessibility and analysis have raised the possibility of producing strong RWE to back FDA regulatory decisions [62]. Researchers now use a nationwide electronic system called the FDA’s Sentinel initiative to monitor the safety of medical products regulated by the agency. The aim of this initiative is to increase the participation of scientists and use new technologies (such as data science and big data), as well as innovative methods for utilizing electronic health records and monitoring drug safety, including other aspects of the use of drugs [63].

More recently on the EMA webpage, we can find a report about ‘Use of RWE in regulatory decision making—EMA publishes review of its studies’ where the agency releases a review of its studies, outlining the steps it has taken to allow for the use of RWD in regulatory decision making, including pharmacovigilance [64].

## 7. Conducting and Reporting Pharmacoepidemiologic Research

ENCePP, in its Guides on Methodological Standards in Pharmacoepidemiology, which is annually updated, discusses new aspects of methods that can be used in different studies to evaluate safety, effectiveness, or drug use [16]. Other organizations have published general guidance on the conduct of pharmacoepidemiology studies such as ISPE Good Pharmacoepidemiology Practices (GPP) that includes data quality issues and archiving [65].

Another important aspect to take into consideration is how all these studies and methodologies that have been described are reported or explained in publications. This is why it is also important to have guidelines for reporting observational studies. The STROBE Statement provides guidance on how to report observational research, with a checklist of items that should be included in articles reporting such research [66]. In addition, the Observational Routinely collected health Data (RECORD) declaration was developed to handle reporting requirements unique to observational studies that make use of regularly gathered health data [67]. STROBE and RECORD have a checklist with relevant items to the title, abstract, introduction, methods, results, and discussion sections of publications, as well as additional details that must be included in these kinds of research reports.

Recently, a joint task force, comprising important international stakeholders, was organized by the International Society for Pharmacoepidemiology and ISPOR—The Professional Society for Health Economics and Outcomes Research—to develop a harmonized protocol template for RWE studies that assess the effect of treatment and are meant to guide decision making. By using a similar text, tabular, and visual format, the HARmonized Protocol Template to Enhance Reproducibility contributes to the development of a shared understanding of desired scientific decisions [68].

The Preferred Reporting Items for Systematic Reviews and Meta-Analyses statement (PRISMA statement) can be used as a basis for reporting systematic reviews with objectives other than evaluating interventions [69]. The MOOSE checklist is another tool used for reporting the meta-analyses of observational studies in epidemiology [70].

The methodological quality of an observational study can be assessed using the NIH Quality Assessment Tools [71] or The Newcastle-Ottawa Scale (NOS) for assessing the quality of nonrandomized studies [72].

To convey transparency and quality in observational studies and to help understand their design and how the study was conducted, Gatto et al. agreed with the existing recommendations, providing guidance on how to use visualizations throughout the study, from its design to the final report, to enable quick, transparent, and evidence-based decision making [73].

## 8. Recommendations

There is a need to assess drug effects (efficacy and safety) in real clinical practice after their commercialization to optimize their use and the management of the patient. Industry, academia, and government should create alliances and work together in generating evidence and making the best decisions for patients.

Depending on our objective, data availability (primary data collection or secondary data), and the research question, we will choose the methodology and the best study design to answer it. When using or choosing a database source for our study, it is important to know the characteristics of the data provided, data validity, as well as their limitations. For this reason, it is important to be sure of data validity and to have information on the data-generating process.

In order to ensure transparency and avoid redundancy in research, two aspects are very important: on the one hand, the registries for observational studies, such as the ENCePP with the EUPASS number (https://www.encepp.eu/encepp/studiesDatabase.jsp, accessed on 23 October 2023), and on the other hand, good quality reporting following the internationally accepted guidelines such as STROBE, RECORD or HARPER. The study reproducibility and the potential source of bias appraisal are important in reporting the results of the studies.

## 9. Conclusions

Pharmacoepidemiology is essential for the evaluation of the real beneficial and harmful effects of medications used massively in large population groups. The increase in large quantities of observational data available from clinical practice has opened a wide range of options for the use of these data for the risk–benefit assessment of post-authorization drugs. This in turn has required the development of new designs and methodologies for the generation of valid evidence, as well as new initiatives to provide guidance and recommendations on how to incorporate RWE into the drug development process. The implication of artificial intelligence will be useful in the management of this data and will open new horizons.

## Figures and Tables

**Table 1:** Main characteristics of observational studies and sources of data used.

Type of Study		Main Utilities	Main Limitations	Main Sources of Data
Descriptive studies	Cross-sectional studies	To provide a snapshot of drug use and its effects on a population	Not good for rare or short-duration diseases.	Any kind of data sources (1), (2), (3), (4), (5), (6), (7), (8), (9), (10), (11)
	Drug-utilization studies	To describe patterns of use of drugs regarding rational use and guidelines	No information on drugs.	(1), (2), (3), (4), (8), (9)
	Ecological studies	To identify patterns of drug use and disease occurrence	Data are inaccurate.	Any kind of population data sources: (2), (3), (4), (5), (6), (8), (9).
Analytical studies	Cohort studies	To study long-term drug effects. Can assess multiple exposures and outcomes.	Need for a large sample size and an extended study period. Not useful for studying rare outcomes or diseases.	Any kind of data sources: (1), (2), (3), (4), (5), (6), (7), (8), (9), (10), (11)
	Case-control studies	To assess rare outcomes or diseases, and those with long latency periods	Accurate selection of control subjects is a challenge. Difficult to find cases.	(1), (2), (3), (4), (5), (6), (9), (10), (11)
	Target trial emulation	Emulates a hypothetical randomized trial Eliminates common sources of bias.	Cannot eliminate the bias that arises from a lack of randomization. Requires detailed data on treatment, outcome, and confounders. Not useful for new drugs.	(1), (2), (3), (4), (8), (9), (11)

(1) Electronic health records (EHRs); (2) prescription databases; (3) pharmacy dispensing databases; (4) disease registries; (5) pharmacovigilance databases; (6) insurance claims databases; (7) national health surveys; (8) economic assessment; (9) patient-generated health data; (10) social media database; (11) other specific registries.

**Table 2:** Description of measures to avoid confounding.

Measures	Description
Propensity score (PS)	Classifies subjects, the covariates of interest
HDPS	Extension of the PS, but using hundreds of covariates
Marginal structural models	Control to time-varying confounders
G-estimation	Control for time-varying confounders
Instrumental variables	To address uncontrolled confounding
Rule-out approach	To deal with unmeasured confounders
Lag-time	To deal with protopathic bias
Negative controls	Identify and resolve confounding and recall bias

HDPS: high-dimensional propensity score.

## Data Availability

No new data were created.
